# Antipsychotic-Related Risks of Type 2 Diabetes Mellitus in Enrollees With Schizophrenia in the National Basic Public Health Service Program in Hunan Province, China

**DOI:** 10.3389/fpsyt.2022.754775

**Published:** 2022-02-24

**Authors:** Feiyun Ouyang, Jun He, Xunjie Cheng, Wei Zhou, Shuiyuan Xiao, Junqun Fang

**Affiliations:** ^1^Department of Social Medicine and Health Management, Xiangya School of Public Health, Central South University, Changsha, China; ^2^Department of Geriatric Medicine, Xiangya Hospital, Central South University, Changsha, China; ^3^Research Center for Public Health and Social Security, School of Public Administration, Hunan University, Changsha, China; ^4^Department of Health Management, Maternal and Child Health Hospital of Hunan Province, Changsha, China

**Keywords:** schizophrenia, antipsychotic, type 2 diabetes mellitus, polytherapy, monotherapy

## Abstract

**Background:**

Antipsychotics contribute to the development of type 2 diabetes mellitus (T2DM) in individuals with schizophrenia. However, the extent of the relationship between antipsychotic use and T2DM varies in different settings, and the magnitude of the drug-specific effects fluctuates widely. This study aimed to explore the association of T2DM with antipsychotic use among enrollees with schizophrenia in China's National Basic Public Health Service Program (NBPHSP) and the drug-specific relationship with T2DM among patients receiving antipsychotic monotherapy.

**Methods:**

We recruited diabetes-free patients with schizophrenia who were enrolled in the NBPHSP of Hunan Province from October 2009 to December 2018. The participants were classified into the following three groups: regular antipsychotic use, intermittent antipsychotic use, and antipsychotic-free groups. The patients were followed up until they received a T2DM diagnosis or until April 2019. Cox regression models were constructed to calculate the overall and drug-specific hazard ratios (HRs) to determine the antipsychotic–T2DM relationship. Interactive and subgroup analyses were performed to assess the heterogeneity of the effects across subgroups.

**Results:**

A total of 122,064 NBPHSP enrollees with schizophrenia were followed up for 1,507,829 cumulative person-years, and 2,313 (1.89%) patients developed T2DM. Patients who regularly and intermittently used antipsychotics had 117% (HR: 2.17, 95% CI: 1.83–2.57) and 53% (HR: 1.53, 95% CI: 1.23–1.90) higher risks of developing T2DM than antipsychotic-free patients, respectively. Regarding monotherapy, the T2DM risk increased by 66, 80, 62, and 64% after the regular use of clozapine, risperidone, chlorpromazine, and perphenazine, respectively. In addition, the antipsychotic-related risk of T2DM decreased as the patient's baseline body mass index, and baseline fasting plasma glucose level, as well as the dietary proportion of animal products, increased.

**Conclusion:**

Antipsychotics, especially clozapine, risperidone, chlorpromazine, and perphenazine, increased the T2DM risk among NBPHSP enrollees with schizophrenia. Mental health officers should accurately identify enrollees at a high risk of T2DM and take appropriate preventive measures to reduce the incidence of T2DM among patients with schizophrenia.

## Introduction

Schizophrenia is a complex mental disorder that incurs a significant burden on patients, their families, and society at large. According to the Global Burden of Disease Study, there were an estimated 13.1 million patients with schizophrenia in 1990, and the number increased to 20.9 million in 2016 (1). Patients with schizophrenia have difficulty achieving both clinical and functional recovery (2), and their life expectancy is ~20% lower than that of the general population (3). Type 2 diabetes mellitus (T2DM) is a common comorbidity in patients with schizophrenia that often results in an increased risk of early death (4, 5). The prevalence of T2DM is 2–3-fold higher among patients with schizophrenia than in the general population (6, 7). The reasons for this high prevalence are multifactorial and are of concern to both researchers and psychiatrists.

As genetic, environmental, and disease-specific factors contribute to the increased risk of T2DM in patients with schizophrenia, the potential role of antipsychotics has been actively evaluated in T2DM onset. Although early evidence has been provided by pharmacoepidemiological studies (8–11), owing to biases and residual confounders, the links identified are weak and inconsistent (11, 12). Over the last decade, results from randomized clinical trials have also indicated the metabolic effects of antipsychotics; however, the duration of most trials was not long enough for diabetes to develop via its natural course (8, 13). Over the past 5 years, some well-designed clinical studies have confirmed the association between antipsychotic use and T2DM development (14). Nevertheless, the association between T2DM and antipsychotic use remains disputable for a number of reasons. First, psychopharmacological management of schizophrenia varies among patients of different racial, ethnic, or cultural backgrounds (15, 16). This results in differences in the duration of antipsychotic treatment and dosage, which may influence antipsychotic-related pro-diabetic effects (10, 17). Second, the risks of T2DM vary with different antipsychotic prescriptions as reported in different studies (13, 18, 19). Finally, different lifestyles and different severities of psychotic symptoms may also lead to inconsistent pro-diabetic effects of antipsychotics; this, however, still requires further verification. Thus, it is important to evaluate the association of T2DM with antipsychotic use in different real-world scenarios.

In China, the number of people with schizophrenia doubled between 1990 and 2010, from 3.09 to 7.16 million (20). In 2004, China heavily invested in the Central Government Support for the Local Management and Treatment of Severe Mental Illnesses Project (21), and individuals with schizophrenia accounted for ~70% of the patients managed (22). In 2009, the project was incorporated into the National Basic Public Health Service Program (NBPHSP) (23), which encompasses 14 categories of public health services, including management of serious mental illness (SMI) and non-communicable diseases (NCDs). As of 2018, the program has managed and treated over four million patients with schizophrenia (22). Further elucidation of the association between antipsychotic use and T2DM among NBPHSP enrollees with schizophrenia in China can provide information for mental health officers to precisely evaluate the T2DM risk among antipsychotic users and accurately identify patients with schizophrenia at a high risk of T2DM. In addition, the results can provide recommendations for antipsychotic selection to mental health officers involved in the NBPHSP.

Therefore, this study was conducted to improve the identification of patients with schizophrenia at a high risk of T2DM in the NBPHSP by exploring (1) the relationship between antipsychotic use and T2DM among patients receiving monotherapy or polytherapy; (2) the drug-specific relationships between antipsychotics and T2DM among patients on monotherapy; and (3) the heterogeneity of the effects of antipsychotics on T2DM development among different subgroups of patients with schizophrenia.

## Subjects and Methods

### Study Population

The present study was based on the SMI and NCDs management systems of the NBPHSP. The SMI management system serves all patients with SMI in a given jurisdiction. Once a patient with SMI is enrolled into the system, a medical team would collect patient information systematically, including socio-demographic characteristics, health-related behaviors, mental condition assessment, and health examination, which allows the establishment of a comprehensive health record. Once patients are enrolled in the system, they are visited by mental health officers every 3 months for risk assessment, mental condition examination, and medication adjustment, if needed. If a patient is out of contact, local administrative officials will help mental health officers to contact the patient and his/her caregivers. In this study, when a patient moved to another province, withdrew from the system, or was completely out of contact, he/she was regarded as lost to follow-up. For those under management, a comprehensive health examination is conducted every 6 months. When patients with elevated blood glucose are identified, they are referred to specialized hospitals for diagnosis and treatment. Those diagnosed with diabetes are registered in the NCDs management system to obtain further medical benefits from the government.

Participants in this study were patients who were diagnosed with schizophrenia and did not have diabetes at the time of diagnosis. Participants were enrolled in the SMI management system from October 2009 to December 2018 in Hunan Province. Patients with schizophrenia in the SMI management system were diagnosed by psychiatrists based on their medical history, clinical symptoms, and course of the disease according to ICD-10 (24). From 2009 to 2018, a total of 181,541 patients with schizophrenia were enrolled in the SMI system in Hunan Province. We excluded 55,374 patients whose records had logical errors, such as the date of the diagnosis earlier than the date of birth or the data of treatment earlier than that of the first episode. Furthermore, 1,556 patients who were diagnosed with diabetes prior to their schizophrenia diagnosis and 2,547 patients without medication information were excluded. Finally, 122,064 patients with schizophrenia were included and followed up in this study.

### Assessment of Antipsychotic Drug Use

Antipsychotic use information was obtained from the SMI management system of the NBPHSP. Although several patients were diagnosed and began antipsychotic therapy before enrolling in the NBPHSP, mental health officers collected the information on the diagnosis, disease condition, and antipsychotic medication for all enrollees *via* medical records or face-to-face interviews with patients and their caregivers at the time of enrollment and during regular follow-up visits.

The participants were classified into the following three groups according to their use of antipsychotic drugs: regular antipsychotic use, intermittent antipsychotic use, and antipsychotic-free groups. Regular antipsychotic use referred to the continuous use of one or more antipsychotic drugs according to medical advice since the initial prescription; intermittent antipsychotic use referred to initial use of the antipsychotic drug(s), which was then discontinued because of patient's poor compliance; antipsychotic-free patients had not taken any antipsychotics since their diagnosis. The follow-up period was defined as the time from the first antipsychotic prescription for the regular and intermittent antipsychotic use groups and from the diagnosis of schizophrenia for the antipsychotic-free group to either the onset of T2DM or April 2019.

Patients who received monotherapy for the entire treatment course were also classified into regular antipsychotic use and intermittent antipsychotic use groups. Considering the sample size and statistical efficacy (25), we only included antipsychotic drugs that were used alone by more than 200 patients in each group, namely, clozapine, risperidone, chlorpromazine, perphenazine, olanzapine, sulpiride, and penfluridol.

### Assessment of T2DM

The outcome of this study was the diagnosis of T2DM, which was obtained from the NCDs management (T2DM) system of the NBPHSP. T2DM was diagnosed by qualified physicians according to any of the following World Health Organization criteria (26, 27): (1) typical symptoms of diabetes (polydipsia, polyuria, polyphagia, and weight loss) and a random blood glucose level ≥ 11.1 mmol/l (200 mg/dl); (2) fasting plasma glucose (FPG) ≥ 7.0 mmol/l (126 mg/dl); (3) plasma glucose level ≥ 11.1 mmol/l (200 mg/dl) in a 2-h oral glucose tolerance test after a 75-g glucose load. Patients without diabetes symptoms were retested on a different day. Diabetes classification was carefully confirmed by physicians according to etiological characteristics and clinical features of the disease at the time of diagnosis.

### Assessment of Covariates

Information on socio-demographic characteristics (age, sex, marital status, educational level, and occupation), health-related behaviors (smoking status, alcohol drinking frequency, diet pattern, and physical activity), and other potential confounding factors (insight and risk ratings) was collected by mental health officers using standardized questionnaires during face-to-face interviews with patients and their caregivers at the time of enrollment.

Three options each were offered to the responders regarding their marital status (unmarried, married, and divorced or widowed), educational level (primary school or below, middle school, and high school or above), and occupation (unemployed, agricultural worker, and other professions). Regarding health-related behaviors, responders were asked about the frequency of their physical activity (never, occasionally, once a week, or every day), alcohol drinking (seldom, occasionally, frequently, or every day), smoking status (non-smoker, current smoker, or former smoker), and diet pattern (plant-based diet, balanced plant- and animal-based diet, or animal-based diet). The insight was also classified into three categories as follows: complete insight meant that patients were aware of their mental disorder, thoroughly recognized the pathological manifestations, and understood the need for treatment; incomplete insight meant that patients were aware of their mental disorder but were unable to correctly recognize and analyze their pathological manifestations, and absent insight meant that patients were unaware of their mental disorder. Risk rating was categorized into three levels as follows: level 0 referred to patients who exhibited no dangerous behavior; level 1 referred to patients who made verbal threats and/or shouted but did not act physically; level 2 or higher referred to patients who exhibited physically abusive behaviors.

At the time of enrollment, the height and weight were measured using a calibrated height and weight gauge. The body mass index (BMI, kg/m^2^) was calculated by dividing weight (kg) by squared height (m). FPG, serum albumin, serum creatinine, and serum nitrogen were tested using standard clinical chemistry methods.

### Data Quality Management

The process of data collection and database management was supervised by full-time staff, and spot checks were conducted according to the work manual. Data were extracted from the database by a statistician under close supervision to ensure data quality. All data sheets were checked and evaluated for completeness after collection.

### Statistical Analysis

The baseline characteristics of the participants were presented as the mean ± standard deviation for continuous variables and as *n* (%) for categorical variables. ANOVA was performed for continuous variables, and a chi-square test was performed for categorical variables to compare differences in baseline characteristics among the groups. Kaplan–Meier survival curves were plotted to determine the timeline of T2DM development in accordance with the use of antipsychotic drugs. The log-rank test was used to compare the T2DM-free rates in the different groups. Three Cox proportional hazard regression models were constructed to calculate the crude and adjusted hazard ratios (HRs) to determine the association between antipsychotic use and T2DM (Model 1: adjusting for age and sex; model 2: adjusting for age, sex, baseline BMI, and baseline FPG; and model 3: adjusting for age, sex, baseline BMI, baseline FPG, family history of diabetes, educational level, occupation, marital status, smoking status, alcohol drinking frequency, diet pattern, physical activity, insight rating, and risk rating). The outcome in the three models was the incidence of T2DM. The time variable was defined as the duration from the first antipsychotic prescription for the regular and intermittent antipsychotic use groups and from the diagnosis of schizophrenia for the antipsychotic-free group to either the onset of T2DM or April 2019. Furthermore, the drug-specific T2DM HRs of different antipsychotics were calculated after adjusting for all variables included in model 3. Interactive and subgroup analyses were conducted to further assess the heterogeneity of the effects across the subgroups. For the sensitivity analysis, we included baseline albumin, creatinine, and nitrogen levels in the Cox regression model to verify the robustness of the results.

Data were analyzed using the SAS statistical package (version 9.4 SAS Institute, Inc., Cary, North Carolina, USA), and graphs were plotted in R (version 4.1.0). All tests were two-sided, and statistical significance was set at *P* < 0.05. In the subgroup analysis, the significant levels were adjusted by the Bonferroni method to minimize the type I error.

## Results

### Population Characteristics

**Figure 1:**
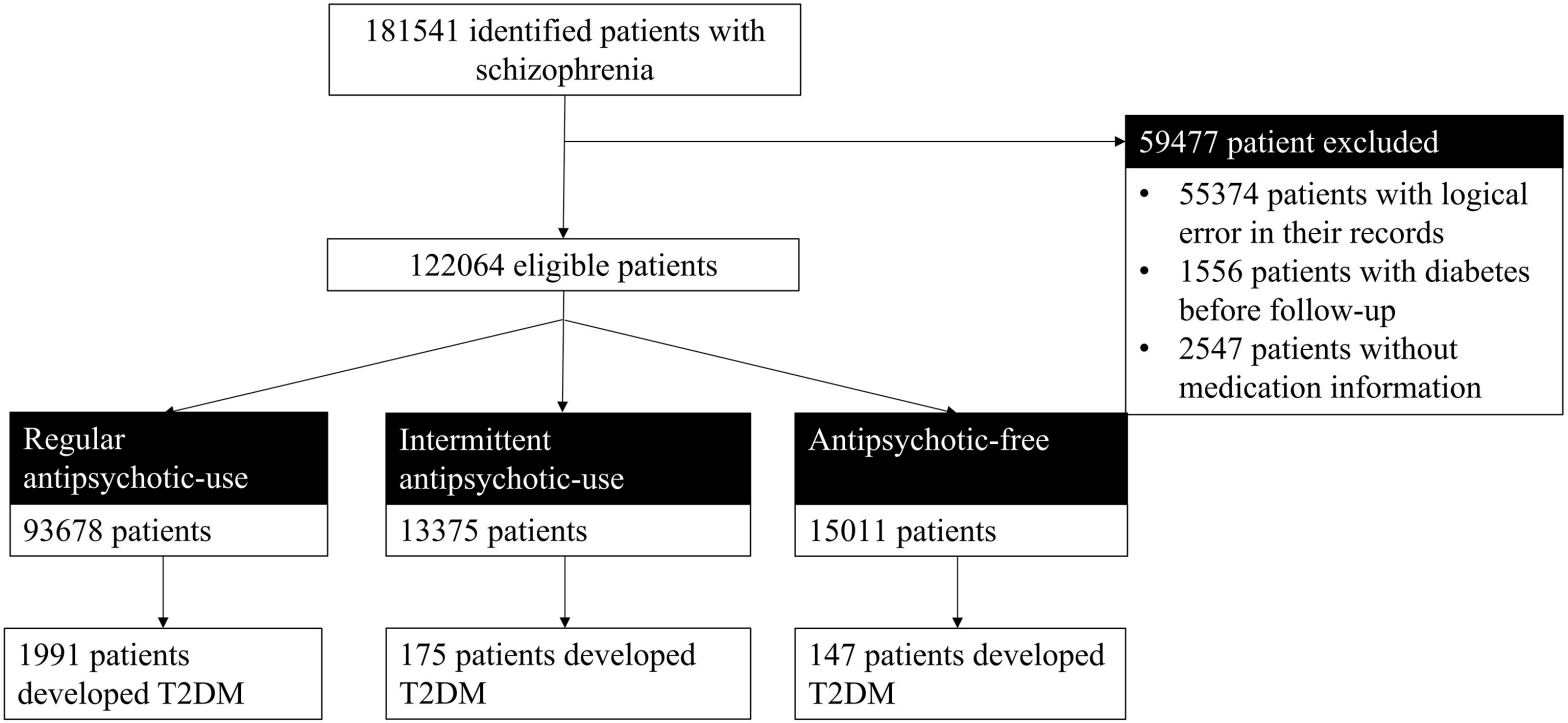
Flowchart of the inclusion of participants. T2DM, type 2 diabetes mellitus.

A total of 122,064 patients with schizophrenia who were under the management of the NBPHSP were followed up for 1,507,829 cumulative person-years. The median duration of the follow-up was 9.16 years (interquartile range: 4.75–18.04 years). Regarding antipsychotic use, 93,678 patients (88.04%) used antipsychotics continuously; 13,375 patients (10.96%) used antipsychotics intermittently, and 15,011 patients (12.30%) were antipsychotic-free (Figure 1).

A summary of the baseline characteristics of the patients is presented in Table 1. The average ages of patients with regular antipsychotic use, those with intermittent antipsychotic use, and antipsychotic-free patients were 33.26 ± 13.26, 35.26 ± 12.61, and 45.48 ± 12.30 years, respectively; the percentages of male patients in the three groups were 49.82%, 48.08%, and 50.5%, respectively; the average baseline BMIs in the three groups were 22.52 ± 2.99, 22.21 ± 2.89, and 22.14 ± 2.82 kg/m^2^, respectively; and the average baseline FPG levels were 5.14 ± 0.61, 5.12 ± 0.60, and 5.13 ± 0.60 mmol/L, respectively. Significant differences were found in all characteristics among the three groups (Table 1).

**Table 1:** Characteristics of participants with different antipsychotic use conditions.

**Variables**	**Regular antipsychotic use M ±SD/N (%)**	**Intermittent antipsychotic use M ±SD/N (%)**	**Antipsychotic free M ±SD/N (%)**	***F*/x^2^**	***P***
Age (years)	33.26 ± 13.26	35.26 ± 12.61	45.48 ± 12.30	348.07	<0.001^*^
**Sex**					
Male	46,669 (49.82)	6,431 (48.08)	7,580 (50.50)	18.33	<0.001^*^
Female	47,009 (50.18)	6,944 (51.92)	7,431 (49.50)		
Baseline BMI (kg/m^2^)	22.52 ± 2.99	22.21 ± 2.89	22.14 ± 2.82	149.00	<0.001^*^
Baseline FPG (mmol/L)	5.14 ± 0.61	5.12 ± 0.60	5.13 ± 0.60	4.37	0.013^*^
**Family history of DM**					
With family history of DM	669 (0.71)	53 (0.40)	71 (0.47)	27.00	<0.001^*^
Without family history of DM	93,009 (99.29)	13,322 (99.60)	14,940 (99.53)		
**Educational level**					
Primary schools or below	38,110 (40.68)	7,023 (52.51)	8,544 (56.92)	1949.00	<0.001^*^
Middle school	40,842 (43.60)	5,044 (37.71)	5,090 (33.91)		
High school or above	14,726 (15.72)	1,308 (9.78)	1,377 (9.17)		
**Occupation**					
Unemployed	1,173 (1.25)	71 (0.53)	109 (0.73)	1331.30	<0.001^*^
Agricultural workers	61,493 (65.64)	10,611 (79.33)	11,180 (74.48)		
Other professions	31,012 (33.10)	2,693 (20.13)	3,722 (24.80)		
**Marital status**					
Unmarried	30,648 (32.72)	3,983 (29.78)	4,592 (30.59)	28.80	<0.001^*^
Married	55,915 (59.69)	8,397 (62.78)	9,123 (60.78)		
Divorced or widowed	7,115 (7.60)	995 (7.44)	1,296 (8.63)		
**Smoking status**					
Non-smoker	84,060 (89.73)	11,930 (89.20)	13,322 (88.75)	19.39	<0.001^*^
Former smoker	508 (0.54)	67 (0.50)	104 (0.69)		
Current smoker	9,110 (9.72)	1,378 (10.30)	1,585 (10.56)		
**Alcohol drinking**					
Never	88,330 (94.29)	12,423 (92.88)	13,848 (92.25)	158.03	<0.001^*^
Occasionally	3,729 (3.98)	614 (4.59)	734 (4.89)		
Frequently	1,071 (1.14)	199 (1.49)	269 (1.79)		
Every day	548 (0.58)	139 (1.04)	160 (1.07)		
**Diet pattern**					
Plant-based diet	6,670 (7.12)	977 (7.30)	1,231 (8.20)	24.00	<0.001^*^
Balanced plant- and animal-based diet	85,392 (91.15)	12,212 (91.30)	13,542 (90.21)		
Animal-based diet	1,616 (1.73)	186 (1.39)	238 (1.59)		
**Physical activity**					
Seldom	8,125 (8.67)	1,080 (8.07)	1,280 (8.53)	172.99	<0.001^*^
Occasionally	1,569 (1.67)	120 (0.90)	136 (0.91)		
Once a week	7,653 (8.17)	896 (6.70)	998 (6.65)		
Every day	76,331 (81.48)	11,279 (84.33)	12,597 (83.92)		
**Insight rating**					
Complete	64,867 (69.24)	6,693 (50.04)	8,347 (55.61)	3163.26	<0.001^*^
Incomplete	27,283 (29.12)	6,192 (46.30)	5,841 (38.91)		
Absent	1,528 (1.63)	490 (3.66)	823 (5.48)		
**Risk rating**					
Level 0	89,737 (95.79)	11,636 (87.00)	13,624 (90.76)	2037.57	<0.001^*^
Level 1	2,972 (3.17)	1,306 (9.76)	1,014 (6.76)		
Level 2 or higher	969 (1.03)	433 (3.24)	373 (2.48)		

* *P < 0.05. M, mean; SD, standard deviation; BMI, body mass index; FPG, fasting plasma glucose; kg, kilogram; m, meter; DM, diabetes mellitus*.

### Effects of Antipsychotics on T2DM Development

In total, 2,313 (1.89%) patients developed diabetes in this cohort. In the regular and intermittent antipsychotic use groups, 1,991 (2.12%) and 175 (1.32%) patients with schizophrenia developed T2DM, respectively. In the antipsychotic-free group, only 147 (0.99%) patients with schizophrenia developed T2DM.

**Figure 2:**
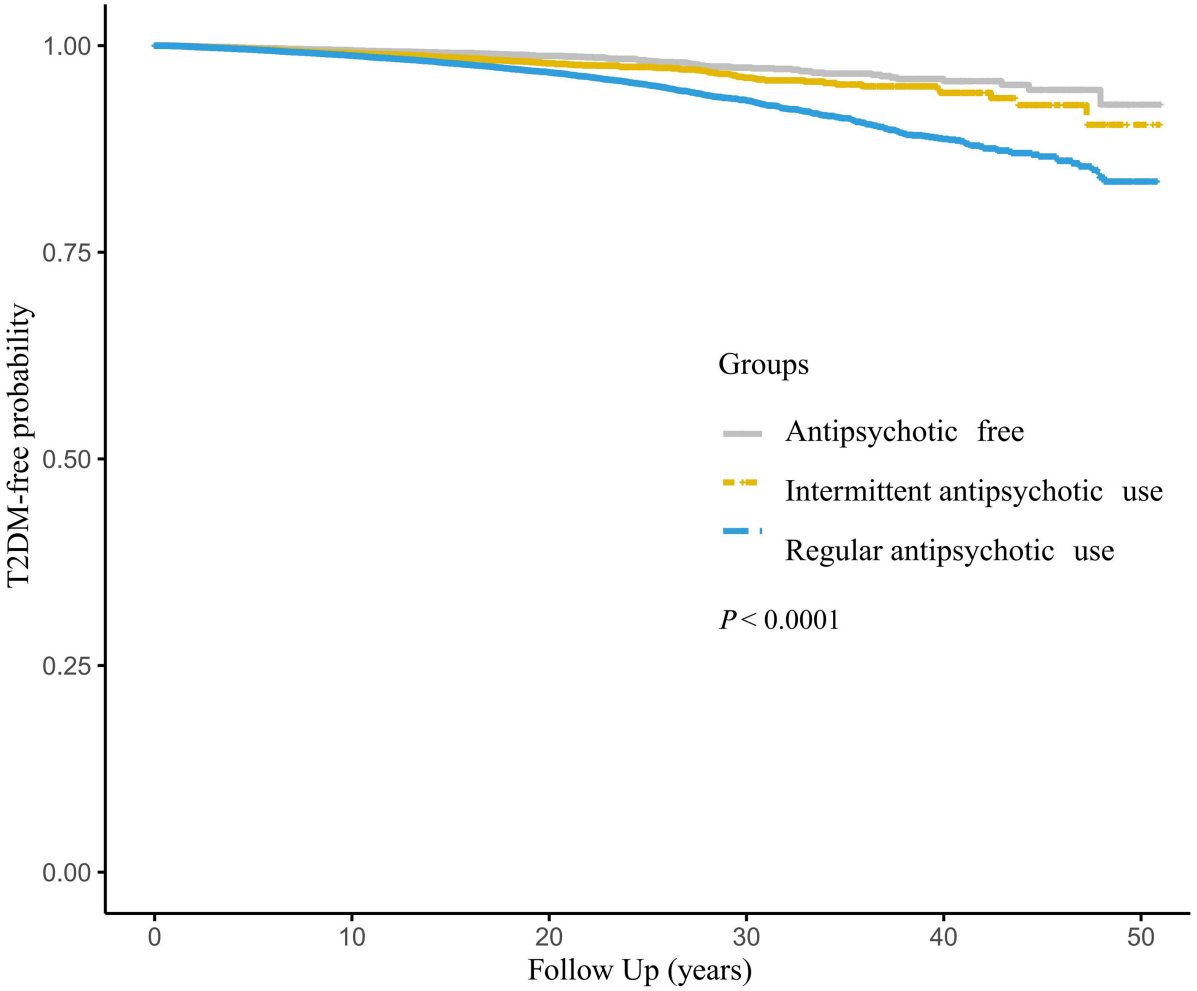
Kaplan–Meier analysis of T2DM by antipsychotic use. T2DM, type 2 diabetes mellitus.

The Kaplan–Meier curves demonstrated the probabilities of being T2DM-free in different groups (Figure 2). There was a significant difference in the probabilities, with a lower probability of being T2DM free associated with an increased frequency of antipsychotics use (log-rank test, *P* < 0.001).

### Hazard Ratio for the Development of T2DM

In model 1, compared with those in the antipsychotic-free group, individuals who continuously used antipsychotics had a 167% higher risk of developing T2DM (HR: 2.67, 95% CI: 2.26–3.16), and individuals who intermittently used antipsychotics had a 52% higher risk of developing T2DM (HR: 1.52, 95% CI: 1.22–1.90). In model 2, individuals who continuously used antipsychotics and those who intermittently used antipsychotics had a 141% (HR: 2.41, 95% CI: 2.04–2.85) and 51% (HR: 1.51, 95% CI: 1.21–1.88) higher risk of developing T2DM than those in the antipsychotic-free group, respectively. In model 3, the risk of developing T2DM was 117% higher in the patients who continuously used antipsychotics (HR: 2.17, 95% CI: 1.83–2.57) and 53% higher in the individuals who intermittently used antipsychotics than in those from the antipsychotic-free group (HR: 1.53, 95% CI: 1.23–1.90) (Table 2).

**Table 2:** Hazard ratios of T2DM according to antipsychotic use.

**Variables**	**Model 1**	**Model 2**	**Model 3**
	**HR (95% CI)**	**HR (95% CI)**	**HR (95% CI)**
Age	1.03 (1.02, 1.03)^*^	1.03 (1.02, 1.03)^*^	1.02 (1.02, 1.03)^*^
**Sex**
Male	Reference	Reference	Reference
Female	1.90 (1.74, 2.08)^*^	1.81 (1.65, 1.97)^*^	1.75 (1.57, 1.94)^*^
**Antipsychotics use**			
Antipsychotic free	Reference	Reference	Reference
Intermittent antipsychotic use	1.52 (1.22, 1.90)^*^	1.51 (1.21, 1.88)^*^	1.53 (1.23, 1.90)^*^
Regular antipsychotic use	2.67 (2.26, 3.16)^*^	2.41 (2.04, 2.85)^*^	2.17 (1.83, 2.57)^*^
Baseline BMI		1.11 (1.10, 1.12)^*^	1.10 (1.09, 1.11)^*^
Baseline FPG		1.66 (1.57, 1.77)^*^	1.66 (1.56, 1.76)^*^
**Family history of diabetes**			
Without family history of DM		Reference	Reference
With family history of DM		4.00 (3.17, 5.04)^*^	3.71 (2.93, 4.70) ^*^
**Marital status**			
Unmarried			Reference
Married			1.38 (1.21, 1.56)^*^
Divorced or widowed			1.13 (0.94, 1.35)
**Educational level**			
Primary school or below			Reference
Middle school			1.05 (0.96, 1.16)
High school or above			1.00 (0.87, 1.14)
**Occupation**			
Unemployed			Reference
Agricultural workers			0.70 (0.52, 0.95)^*^
Other professions			0.82 (0.60, 1.10)
**Smoking status**			
Non-smoker			Reference
Former smoker			1.00 (0.57, 1.74)
Current smoker			1.22 (1.02, 1.45)^*^
**Alcohol drinking**			
Never			Reference
Occasionally			0.95 (0.74, 1.21)
Frequently			0.97 (0.63, 1.51)
Every day			1.18 (0.69, 2.03)
**Diet pattern**			
Plant-based diet			Reference
Balanced plant- and animal-based diet			0.84 (0.72, 0.97)^*^
Animal-based diet			0.86 (0.61, 1.20)
**Physical activity**			
Seldom			Reference
Occasionally			0.92 (0.69, 1.22)
Once a week			0.92 (0.78, 1.09)
Every day			0.70 (0.61, 0.79)^*^
**Insight rating**			
Complete			Reference
Incomplete			0.67 (0.60, 0.74)^*^
Absent			0.56 (0.39, 0.81)^*^
**Risk rating**			
Level 0			Reference
Level 1			1.11 (0.87, 1.42)
Level 2 or higher			0.76 (0.44, 1.32)

* *P < 0.05. DM, diabetes mellitus; T2DM, type 2 diabetes mellitus; HR, hazard ratio; CI, confidence interval; BMI, body mass index; FPG, fasting plasma glucose*.

**Figure 3:**
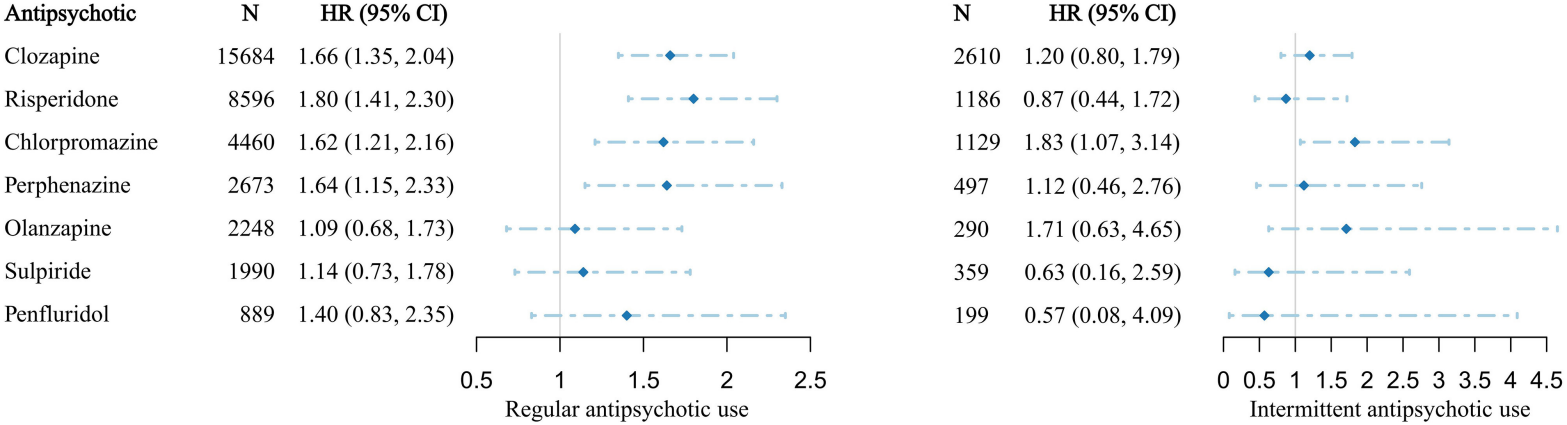
Antipsychotic-related hazard ratios of T2DM in patients with schizophrenia receiving monotherapy. HR, hazard ratio; CI, confidence interval. HRs and 95% CIs were calculated by multivariate-adjusted Cox regression model.

With regard to monotherapy, the risk for developing T2DM increased following the regular use of clozapine by 66% (HR: 1.66, 95% CI: 1.35–2.04), risperidone by 80% (HR: 1.80, 95% CI: 1.41–2.30), chlorpromazine by 62% (HR: 1.62, 95% CI: 1.21–2.16), and perphenazine by 64% (HR: 1.64, 95% CI: 1.15–2.33). The T2DM hazard ratios were not statistically significant after the regular use of olanzapine (HR: 1.09, 95% CI: 0.68–1.73), sulpiride (HR: 1.14, 95% CI: 0.73–1.78), and penfluridol (HR: 1.40, 95% CI: 0.83–2.35) (Figure 3).

### Interactive, Subgroup, and Sensitivity Analyses

In the interactive analysis, the effects of interactions between antipsychotic use and the baseline BMI, baseline FPG level, and diet pattern on the development of T2DM were statistically significant (*P* < 0.05). None of the interactions between other factors (age, sex, alcohol drinking, smoking status, physical activity, insight rating, risk rating, and family history of diabetes) and antipsychotic use on the development of T2DM were statistically significant. In the subgroup analysis, the risk of T2DM with antipsychotic use decreased as the baseline BMI and baseline FPG level, as well as the proportion of animal products in the patient's diet, increased (Supplementary Table 1). Similar trends were observed for patients treated with monotherapy (Supplementary Figure 1). In the sensitivity analysis, the results were consistent with those of models 1–3 (Supplementary Table 2).

## Discussion

### Main Findings

In the present study, we found that regular antipsychotic use was associated with a 117% higher risk of developing T2DM among NBPHSP enrollees with schizophrenia. Regarding monotherapy, the T2DM risk increased by 66%, 80%, 62% and 64% after the regular use of clozapine, risperidone, chlorpromazine, and perphenazine, respectively. The risk of developing T2DM due to antipsychotic use decreased with an increase in the patient's baseline BMI and baseline FPG level, as well as the proportion of animal products in the diet. The strengths of this study include a large sample size and a long follow-up period based on the NBPHSP data in China, as well as a comprehensive exploration of the heterogeneity of the effects of antipsychotics on T2DM development among different subgroups of patients. This study provides information for identifying patients at a high risk of T2DM in the SMI system of the NBPHSP and for developing strategies to prevent the development of T2DM among patients with antipsychotic prescriptions.

### Comparison With Similar Studies and Interpretations

In this study, we found that patients with schizophrenia who regularly or intermittently took antipsychotics had a significantly higher risk of developing T2DM than antipsychotic-free patients, which was consistent with the results of previous studies (7, 19, 28–30). The T2DM risk was higher in antipsychotic users than in antipsychotic-free controls (10, 19, 30), for which there are several potential reasons. For instance, the association between antipsychotics and diabetes is believed to be mediated by weight gain (31, 32). The increased risk of diabetes may also be associated with insulin resistance, reduced insulin sensitivity, and abnormal blood glucose regulation, which is thought to be mediated by muscarinic M3 receptor antagonism (33).

Regarding monotherapy, patients who regularly used clozapine, risperidone, chlorpromazine, and perphenazine had a significantly higher risk of developing T2DM than antipsychotic-free patients. According to previous studies, the pro-diabetic effects differ for different antipsychotics, and the extent of this risk varies widely (13, 19). Some studies have reported a higher prevalence of T2DM among users of all categories of antipsychotic medications than among antipsychotic-free participants (7, 28). In particular, clozapine and olanzapine were identified as the top two diabetes-causing agents (34, 35). However, the relationship of diabetes with olanzapine use was found to be insignificant in two population-based studies (10, 19). The olanzapine-caused increase in blood sugar levels can be reversed by discontinuing olanzapine (36). Since olanzapine demonstrated a stronger obesogenic effect than that of most other antipsychotics (31, 37), we cannot rule out the possibility that some olanzapine users switched to other antipsychotics or stopped using antipsychotic drugs entirely because of the significant weight gain (38), which may have resulted in the insignificant relationship between olanzapine and T2DM in this study.

In this study, the risk of antipsychotic-related T2DM decreased as the patient's baseline BMI and baseline FPG increased, which may be attributed to the efforts of mental health staff in the NBPHSP. As reported in previous studies, patients with a high baseline BMI and FPG level were at a higher risk of diabetes (39, 40). Regarding those with a high risk of developing T2DM, according to the recommendation in the *Chinese Schizophrenia Prevention and Control Guideline* (41) and the requirement in the *National Basic Public Health Service Specifications* (42), psychiatrists should initiate a lifestyle intervention at the beginning of treatment, helping patients adjust their diets and maintain physical activity. In addition, psychiatrists were requested to try to choose antipsychotics with fewer metabolic side effects and prescribe medications that help prevent diabetes, such as metformin or orlistat, when necessary (43). Furthermore, an increase in BMI, regardless of the baseline BMI, is an independent risk factor of diabetes (44). In the case of a relatively high BMI and FPG level, psychiatrists, caregivers, and patients would make efforts to keep the BMI and FPG stable, which could buffer the pro-diabetic effects of antipsychotics, to some extent.

The association between antipsychotics and T2DM in patients on balanced plant- and animal-based or animal-based diets was lower than that in patients on a plant-based diet. People with schizophrenia often have a low socioeconomic status and income (45), which makes it difficult for them to obtain high-quality food. Although plant-based diets have been proven to be protective against diabetes, low-quality plant-based diets may lead to protein deficiency in patients with schizophrenia, making it difficult for the body to maintain homeostasis in response to the metabolic side effects of antipsychotics (46). A high-quality diet with an adequate amount of protein is effective in improving blood glucose control (39). As recommended by the Canadian Diabetes Association in 2013, both the quantity and quality of protein intake must be optimized to meet the requirements for essential amino acids to prevent the onset of T2DM (47). In addition, patients whose diets include a high proportion of animal products consume more heme iron (48). Iron constitutes the metal nucleus of many cellular enzymes and is important for most cellular processes, including β-cell metabolism and insulin secretion (49), which help in glycemic regulation. Hence, a proper diet with sufficient animal products and adequate high-quality plant foods might help mitigate the pro-diabetic side effects of antipsychotics.

### Implications of the Findings for the Prevention and Treatment of T2DM

There are several practical implications of our findings. First, mental health officers involved in the NBPHSP should provide dietary and behavioral guidance, along with antipsychotic prescriptions, to patients with schizophrenia to help reduce T2DM risks (50). Second, mental health officers should conduct a regular blood glucose screening of patients with schizophrenia, especially those who regularly use antipsychotics. With a timely risk assessment and comprehensive intervention strategy, mental health officers and caregivers can help patients with schizophrenia reduce the risk of T2DM, which also reduces the risks of secondary cardiovascular disease and the associated mortality (50). Third, ensuring adequate amounts of high-quality animal products in the diet can help patients maintain glucometabolic homeostasis in response to antipsychotics. Lastly, the existing preventive measures for patients at a high risk of diabetes are effective to some extent. However, further implementation of these measures is needed, along with more attention to groups with a lower risk of diabetes.

### Limitations and Future Research

There are several limitations to this study. First, the assessment of antipsychotic drug use was only based on medical records and face-to-face interviews, but it was not confirmed by serum level measurements. To avoid potential information bias, we should consider serum level measurements of antipsychotic medications in future research. Second, the dosage of an antipsychotic drug affects the risk of developing T2DM (51); however, detailed information on the dosages was not collected in this program, which made our results only a rough estimate. Third, information related to T2DM diagnosis was obtained from the NCDs management system of the NBPHSP. Since the proportion of antipsychotic users with undiagnosed diabetes was considerable (52), this study inevitably underestimated the incidence of T2DM. In addition, patients with schizophrenia who do not require medication may have less connection with the system. This may lead to under-diagnosis of T2DM in the antipsychotic-free group and, therefore, exaggerate the effects of antipsychotics on the development of T2DM. Fourth, we were unable to control some potential confounders, such as a sedentary lifestyle, because of the lack of information. Thus, residual confounding factors may have influenced the study findings. Lastly, the antipsychotic-induced weight gain or blood glucose increase might lead patients to discontinue therapy, which would cause an inevitable survival bias (38). Despite these limitations, our findings revealed the actual risks of antipsychotics-induced T2DM in real-world practice.

It is crucial to conduct further research to prevent T2DM among antipsychotic users. The contribution of various known risk factors, such as a BMI change and diet pattern, to the association of T2DM with antipsychotic use warrants further exploration. In addition, antipsychotics with fewer metabolic side effects are yet to be developed. In the selection of the treatment strategy, efficacy should be the most important consideration. Further studies should focus on exploring optimal treatment strategies that balance both therapeutic efficacy and metabolic risk management not only in the NBPHSP but also in different scenarios.

## Conclusions

Antipsychotics, especially clozapine, risperidone, chlorpromazine, and perphenazine, increased the risk of T2DM in enrollees with schizophrenia in the NBPHSP. However, the risk decreased as the patient's baseline BMI and baseline FPG level, as well as the proportion of animal products in the patient's diet, increased. For patients at a high risk of developing T2DM, mental health workers should provide regular assessments of glucose levels, recommend lifestyle modifications, and adjust medication prescriptions when necessary.

## Data Availability Statement

The generated datasets are available after the approval of National Basic Public Health Service Program of Hunan Province, China. Introduction to the program and detailed information of the application can be found in http://www.nbphsp.org.cn/. Further inquiries can be directed to the corresponding author/s.

## Ethics Statement

This study involving human participants was reviewed and approved by the institutional review board of Xiangya School of Public Health, Central South University (NO. XYGW-2021-41).

## Author Contributions

FO, SX, and JF contributed to the conception and design of the study. FO and JH performed the statistical analysis and wrote the first draft of the manuscript. WZ, XC, and SX reviewed and edited the manuscript. All authors read and approved the final manuscript.

## Funding

This work was supported by the National Key Research and Development Program of China (Grant No: 2016YFC0900802).

## Conflict of Interest

The authors declare that the research was conducted in the absence of any commercial or financial relationships that could be construed as a potential conflict of interest.

## Publisher's Note

All claims expressed in this article are solely those of the authors and do not necessarily represent those of their affiliated organizations, or those of the publisher, the editors and the reviewers. Any product that may be evaluated in this article, or claim that may be made by its manufacturer, is not guaranteed or endorsed by the publisher.
