# Supplementary material for: Antipsychotic-Related Risks of Type 2 Diabetes Mellitus in Enrollees With Schizophrenia in the National Basic Public Health Service Program in Hunan Province, China
Source: Front Psychiatry. 2022 Feb 24;13:754775. doi: 10.3389/fpsyt.2022.754775 (PMC8909132; doi:10.3389/fpsyt.2022.754775)
Supplement: Supplementary file 1 [file Data_Sheet_1.docx]

***Supplemental Material***

**1. Supplemental material table**

Table S1. Interactive and subgroup analyses

| **Subgroups** | **Variable** | **Estimate** | **SE** | ***P*** | **HR** | **95% CI** | | ***P* for**  **interaction** |
| --- | --- | --- | --- | --- | --- | --- | --- | --- |
|  |  |  |  |  |  | **Lower** | **Upper** |  |
| Age |  |  |  |  |  |  |  | 0.893 |
| ≤36 | Antipsychotic free | Reference |  |  |  |  |  |  |
|  | Intermittent antipsychotic use | -1.69 | 1.08 | 0.119 | 0.19 | 0.02 | 1.54 |  |
|  | Regular antipsychotic use | 0.81 | 0.42 | 0.055 | 2.24 | 0.98 | 5.13 |  |
| >36 and ≤46 | Antipsychotic free | Reference |  |  |  |  |  |  |
|  | Intermittent antipsychotic use | 0.46 | 0.29 | 0.121 | 1.58 | 0.89 | 2.81 |  |
|  | Regular antipsychotic use | 0.76 | 0.23 | 0.001^c^ | 2.14 | 1.36 | 3.37 |  |
| >46 and ≤55 | Antipsychotic free | Reference |  |  |  |  |  |  |
|  | Intermittent antipsychotic use | 0.37 | 0.20 | 0.068 | 1.45 | 0.97 | 2.15 |  |
|  | Regular antipsychotic use | 0.69 | 0.16 | <0.001^c^ | 2.00 | 1.47 | 2.72 |  |
| >55 | Antipsychotic free | Reference |  |  |  |  |  |  |
|  | Intermittent antipsychotic use | 0.51 | 0.16 | 0.001^c^ | 1.67 | 1.23 | 2.26 |  |
|  | Regular antipsychotic use | 0.81 | 0.12 | <0.001^c^ | 2.25 | 1.78 | 2.85 |  |
| Sex |  |  |  |  |  |  |  | 0.900 |
| Male | Antipsychotic free | Reference |  |  |  |  |  |  |
|  | Intermittent antipsychotic use | 0.23 | 0.21 | 0.258 | 1.26 | 0.84 | 1.90 |  |
|  | Regular antipsychotic use | 0.65 | 0.15 | <0.001^a^ | 1.91 | 1.43 | 2.57 |  |
| Female | Antipsychotic free | Reference |  |  |  |  |  |  |
|  | Intermittent antipsychotic use | 0.51 | 0.13 | <0.001^a^ | 1.67 | 1.28 | 2.17 |  |
|  | Regular antipsychotic use | 0.84 | 0.11 | <0.001^a^ | 2.32 | 1.89 | 2.85 |  |
| Baseline BMI |  |  |  |  |  |  |  | 0.007^*^ |
| <18.5 | Antipsychotic free | Reference |  |  |  |  |  |  |
|  | Intermittent antipsychotic use | 1.99 | 0.77 | 0.010^b^ | 7.31 | 1.61 | 33.18 |  |
|  | Regular antipsychotic use | 1.57 | 0.73 | 0.032 | 4.81 | 1.14 | 20.25 |  |
| 18.5-23.9 | Antipsychotic free | Reference |  |  |  |  |  |  |
|  | Intermittent antipsychotic use | 0.49 | 0.14 | <0.001^b^ | 1.63 | 1.24 | 2.15 |  |
|  | Regular antipsychotic use | 0.88 | 0.11 | <0.001^b^ | 2.40 | 1.94 | 2.97 |  |
| ≥24 | Antipsychotic free | Reference |  |  |  |  |  |  |
|  | Intermittent antipsychotic use | 0.13 | 0.20 | 0.509 | 1.14 | 0.77 | 1.69 |  |
|  | Regular antipsychotic use | 0.56 | 0.15 | <0.001^b^ | 1.74 | 1.31 | 2.32 |  |
| Baseline FPG |  |  |  |  |  |  |  | 0.029^*^ |
| Quantile 1 | Antipsychotic free | Reference |  |  |  |  |  |  |
|  | Intermittent antipsychotic use | 0.49 | 0.22 | 0.029 | 1.63 | 1.05 | 2.52 |  |
|  | Regular antipsychotic use | 0.92 | 0.17 | <0.001^b^ | 2.51 | 1.79 | 3.54 |  |
| Quantile 2 | Antipsychotic free | Reference |  |  |  |  |  |  |
|  | Intermittent antipsychotic use | 0.50 | 0.21 | 0.018 | 1.65 | 1.09 | 2.50 |  |
|  | Regular antipsychotic use | 0.81 | 0.17 | <0.001^b^ | 2.25 | 1.63 | 3.12 |  |
| Quantile 3 | Antipsychotic free | Reference |  |  |  |  |  |  |
|  | Intermittent antipsychotic use | 0.35 | 0.16 | 0.033 | 1.42 | 1.03 | 1.96 |  |
|  | Regular antipsychotic use | 0.70 | 0.12 | <0.001^b^ | 2.00 | 1.57 | 2.56 |  |
| Marital status |  |  |  |  |  |  |  | 0.723 |
| Unmarried | Antipsychotic free | Reference |  |  |  |  |  |  |
|  | Intermittent antipsychotic use | 0.65 | 0.32 | 0.046 | 1.91 | 1.01 | 3.60 |  |
|  | Regular antipsychotic use | 1.01 | 0.25 | <0.001^b^ | 2.75 | 1.67 | 4.51 |  |
| Married | Antipsychotic free | Reference |  |  |  |  |  |  |
|  | Intermittent antipsychotic use | 0.40 | 0.12 | 0.001^b^ | 1.50 | 1.17 | 1.91 |  |
|  | Regular antipsychotic use | 0.72 | 0.10 | <0.001^b^ | 2.06 | 1.70 | 2.49 |  |
| Divorced or widowed | Antipsychotic free | Reference |  |  |  |  |  |  |
|  | Intermittent antipsychotic use | 0.25 | 0.44 | 0.579 | 1.28 | 0.54 | 3.05 |  |
|  | Regular antipsychotic use | 0.94 | 0.30 | 0.002^b^ | 2.55 | 1.41 | 4.63 |  |
| Educational level |  |  |  |  |  |  |  | 0.361 |
| Primary school or below | Antipsychotic free | Reference |  |  |  |  |  |  |
|  | Intermittent antipsychotic use | 0.55 | 0.14 | <0.001^b^ | 1.74 | 1.31 | 2.31 |  |
|  | Regular antipsychotic use | 0.83 | 0.11 | <0.001^b^ | 2.30 | 1.84 | 2.87 |  |
| Middle school | Antipsychotic free | Reference |  |  |  |  |  |  |
|  | Intermittent antipsychotic use | 0.29 | 0.21 | 0.152 | 1.34 | 0.90 | 2.01 |  |
|  | Regular antipsychotic use | 0.73 | 0.16 | <0.001^b^ | 2.07 | 1.52 | 2.81 |  |
| High school or above | Antipsychotic free | Reference |  |  |  |  |  |  |
|  | Intermittent antipsychotic use | -0.05 | 0.38 | 0.902 | 0.95 | 0.45 | 2.00 |  |
|  | Regular antipsychotic use | 0.45 | 0.25 | 0.075 | 1.56 | 0.96 | 2.56 |  |
| Occupation |  |  |  |  |  |  |  | 0.382 |
| Unemployed | Antipsychotic free | Reference |  |  |  |  |  |  |
|  | Intermittent antipsychotic use | -16.71 | 4011.54 | 0.997 | 0.00 | - | - |  |
|  | Regular antipsychotic use | 0.44 | 0.72 | 0.541 | 1.56 | 0.38 | 6.44 |  |
| Agricultural workers | Antipsychotic free | Reference |  |  |  |  |  |  |
|  | Intermittent antipsychotic use | 0.50 | 0.13 | <0.001^b^ | 1.65 | 1.28 | 2.13 |  |
|  | Regular antipsychotic use | 0.75 | 0.10 | <0.001^b^ | 2.12 | 1.72 | 2.60 |  |
| Other professions | Antipsychotic free | Reference |  |  |  |  |  |  |
|  | Intermittent antipsychotic use | 0.16 | 0.23 | 0.494 | 1.17 | 0.74 | 1.86 |  |
|  | Regular antipsychotic use | 0.80 | 0.16 | <0.001^b^ | 2.22 | 1.63 | 3.03 |  |
| Alcohol drinking |  |  |  |  |  |  |  | 0.528 |
| Never | Antipsychotic free | Reference |  |  |  |  |  |  |
|  | Intermittent antipsychotic use | 0.37 | 0.12 | 0.002^c^ | 1.44 | 1.15 | 1.81 |  |
|  | Regular antipsychotic use | 0.77 | 0.09 | <0.001^c^ | 2.15 | 1.81 | 2.56 |  |
| Occasionally | Antipsychotic free | Reference |  |  |  |  |  |  |
|  | Intermittent antipsychotic use | 0.71 | 0.55 | 0.191 | 2.04 | 0.70 | 5.96 |  |
|  | Regular antipsychotic use | 0.78 | 0.43 | 0.072 | 2.18 | 0.93 | 5.09 |  |
| Frequently | Antipsychotic free | Reference |  |  |  |  |  |  |
|  | Intermittent antipsychotic use | 1.91 | 1.12 | 0.087 | 6.76 | 0.76 | 60.55 |  |
|  | Regular antipsychotic use | 1.26 | 1.05 | 0.229 | 3.54 | 0.45 | 27.76 |  |
| Every day | Antipsychotic free | Reference |  |  |  |  |  |  |
|  | Intermittent antipsychotic use | 2.10 | 0.61 | <0.001^c^ | 8.20 | 2.48 | 27.08 |  |
|  | Regular antipsychotic use | 1.58 | 0.56 | 0.005^c^ | 4.87 | 1.61 | 14.71 |  |
| Smoking status |  |  |  |  |  |  |  | 0.497 |
| Nonsmoker | Antipsychotic free | Reference |  |  |  |  |  |  |
|  | Intermittent antipsychotic use | 0.46 | 0.12 | <0.001^b^ | 1.59 | 1.26 | 2.00 |  |
|  | Regular antipsychotic use | 0.78 | 0.09 | <0.001^b^ | 2.19 | 1.83 | 2.62 |  |
| Former smoker | Antipsychotic free | Reference |  |  |  |  |  |  |
|  | Intermittent antipsychotic use | 0.52 | 0.79 | 0.510 | 1.68 | 0.36 | 7.84 |  |
|  | Regular antipsychotic use | -0.23 | 0.67 | 0.730 | 0.79 | 0.21 | 2.94 |  |
| Current smoker | Antipsychotic free | Reference |  |  |  |  |  |  |
|  | Intermittent antipsychotic use | 0.02 | 0.40 | 0.954 | 1.02 | 0.47 | 2.22 |  |
|  | Regular antipsychotic use | 0.74 | 0.28 | 0.008 | 2.10 | 1.21 | 3.64 |  |
| Dietary pattern |  |  |  |  |  |  |  | 0.006^*^ |
| Plant-based diet | Antipsychotic free | Reference |  |  |  |  |  |  |
|  | Intermittent antipsychotic use | 1.12 | 0.46 | 0.015 | 3.08 | 1.24 | 7.65 |  |
|  | Regular antipsychotic use | 1.59 | 0.39 | <0.001^b^ | 4.89 | 2.28 | 10.47 |  |
| Balanced plant- and animal-  based diet | Antipsychotic free | Reference |  |  |  |  |  |  |
|  | Intermittent antipsychotic use | 0.37 | 0.12 | 0.001^b^ | 1.45 | 1.15 | 1.83 |  |
|  | Regular antipsychotic use | 0.72 | 0.09 | <0.001^b^ | 2.06 | 1.73 | 2.46 |  |
| Animal-based diet | Antipsychotic free | Reference |  |  |  |  |  |  |
|  | Intermittent antipsychotic use | 0.19 | 0.73 | 0.794 | 1.21 | 0.29 | 5.06 |  |
|  | Regular antipsychotic use | 0.10 | 0.56 | 0.859 | 1.10 | 0.37 | 3.28 |  |
| Physical activity |  |  |  |  |  |  |  | 0.090 |
| Never | Antipsychotic free | Reference |  |  |  |  |  |  |
|  | Intermittent antipsychotic use | -0.02 | 0.32 | 0.958 | 0.98 | 0.53 | 1.83 |  |
|  | Regular antipsychotic use | 0.44 | 0.22 | 0.047 | 1.55 | 1.01 | 2.39 |  |
| Occasionally | Antipsychotic free | Reference |  |  |  |  |  |  |
|  | Intermittent antipsychotic use | -0.82 | 0.78 | 0.298 | 0.44 | 0.10 | 2.06 |  |
|  | Regular antipsychotic use | -0.31 | 0.49 | 0.521 | 0.73 | 0.28 | 1.90 |  |
| Once a week | Antipsychotic free | Reference |  |  |  |  |  |  |
|  | Intermittent antipsychotic use | 0.93 | 0.39 | 0.016 | 2.52 | 1.18 | 5.37 |  |
|  | Regular antipsychotic use | 0.98 | 0.31 | 0.002^c^ | 2.67 | 1.45 | 4.92 |  |
| Every day | Antipsychotic free | Reference |  |  |  |  |  |  |
|  | Intermittent antipsychotic use | 0.48 | 0.13 | <0.001^c^ | 1.61 | 1.25 | 2.07 |  |
|  | Regular antipsychotic use | 0.84 | 0.10 | <0.001^c^ | 2.33 | 1.91 | 2.84 |  |
| Insight rating |  |  |  |  |  |  |  | 0.065 |
| Complete | Antipsychotic free | Reference |  |  |  |  |  |  |
|  | Intermittent antipsychotic use | 0.24 | 0.14 | 0.081 | 1.27 | 0.97 | 1.66 |  |
|  | Regular antipsychotic use | 0.64 | 0.10 | <0.001^b^ | 1.89 | 1.56 | 2.29 |  |
| Incomplete | Antipsychotic free | Reference |  |  |  |  |  |  |
|  | Intermittent antipsychotic use | 0.83 | 0.21 | <0.001^b^ | 2.28 | 1.50 | 3.47 |  |
|  | Regular antipsychotic use | 1.09 | 0.19 | <0.001^b^ | 2.98 | 2.07 | 4.29 |  |
| absent | Antipsychotic free | Reference |  |  |  |  |  |  |
|  | Intermittent antipsychotic use | 0.49 | 0.83 | 0.559 | 1.63 | 0.32 | 8.28 |  |
|  | Regular antipsychotic use | 1.44 | 0.63 | 0.022 | 4.22 | 1.23 | 14.45 |  |
| Risk rating |  |  |  |  |  |  |  | 0.165 |
| Level 0 | Antipsychotic free | Reference |  |  |  |  |  |  |
|  | Intermittent antipsychotic use | 0.41 | 0.12 | <0.001^b^ | 1.51 | 1.20 | 1.89 |  |
|  | Regular antipsychotic use | 0.74 | 0.09 | <0.001^b^ | 2.10 | 1.77 | 2.50 |  |
| Level 1 | Antipsychotic free | Reference |  |  |  |  |  |  |
|  | Intermittent antipsychotic use | 0.79 | 0.53 | 0.137 | 2.20 | 0.78 | 6.23 |  |
| Level 2 or higher | Regular antipsychotic use | 1.35 | 0.48 | 0.005^b^ | 3.88 | 1.52 | 9.88 |  |
|  | Antipsychotic free | Reference |  |  |  |  |  |  |
|  | Intermittent antipsychotic use | 0.12 | 0.78 | 0.877 | 1.13 | 0.24 | 5.22 |  |
|  | Regular antipsychotic use | 1.19 | 0.67 | 0.074 | 3.29 | 0.89 | 12.14 |  |
| Family history of DM |  |  |  |  |  |  |  | 0.931 |
| Without family history of DM | Antipsychotic free | Reference |  |  |  |  |  |  |
|  | Intermittent antipsychotic use | 0.42 | 0.11 | <0.001^a^ | 1.53 | 1.22 | 1.90 |  |
|  | Regular antipsychotic use | 0.77 | 0.09 | <0.001^a^ | 2.16 | 1.82 | 2.56 |  |
| With family history of DM | Antipsychotic free | Reference |  |  |  |  |  |  |
|  | Intermittent antipsychotic use | 0.80 | 1.04 | 0.442 | 2.23 | 0.29 | 17.25 |  |
|  | Regular antipsychotic use | 1.30 | 0.73 | 0.007 | 3.65 | 0.87 | 15.35 |  |

Note: ^*^The significance level for interaction was 0.05; ^a^The Bonferroni-corrected significance levels for sex and family history of DM were 0.05/2=0.025; ^b^The Bonferroni-corrected significance levels for baseline BMI, baseline FPG, marital status, education level, occupation, smoking status, dietary pattern, insight, and risk rating were 0.05/3=0.017; ^c^The Bonferroni-corrected significance levels for age, alcohol drinking, and physical activity were 0.05/4=0.0125. SE=standard error; HR=hazard ratio; CI= confidence interval; FPG = fasting plasma glucose; DM=diabetes mellitus; BMI= body mass index.

**2. Supplemental material figure**


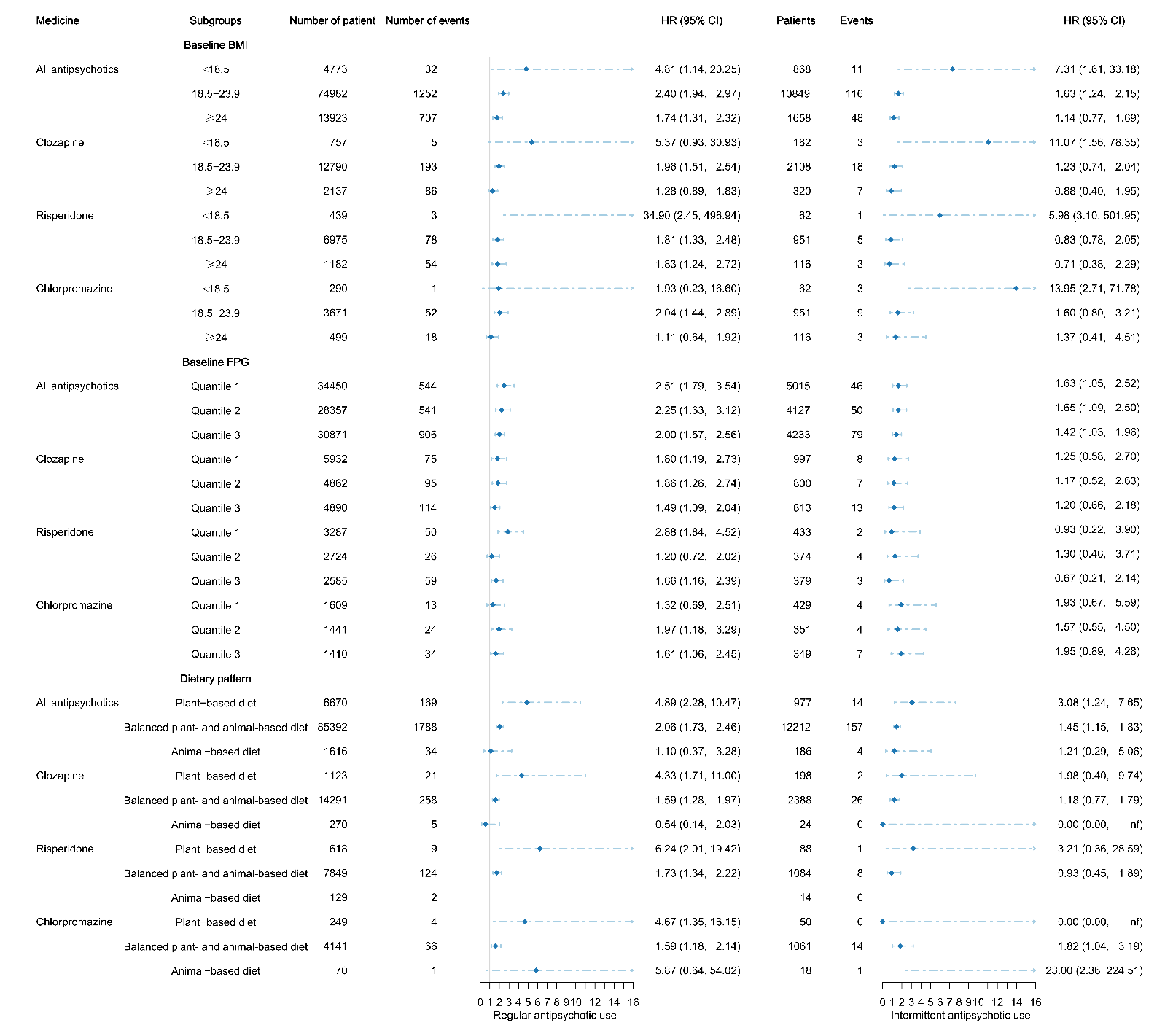


Figure S1. Subgroup analysis based on the insight of schizophrenia patients

Note: HR=hazard ratio; CI= confidence interval; BMI= body mass index; FPG= fasting plasma glucose

**3. Supplemental material table 2**

Table S2. Sensitivity analysis

| **Variable** | **Estimate** | **SE** | **HR** | **Lower** | **Upper** | ***P* value** |
| --- | --- | --- | --- | --- | --- | --- |
| Age | 0.02 | <0.01 | 1.03 | 1.02 | 1.03 | <0.001^*^ |
| Sex |  |  |  |  |  |  |
| Male | Reference |  |  |  |  |  |
| Female | 0.61 | 0.07 | 1.85 | 1.59 | 2.14 | <0.001^*^ |
| Antipsychotics-use |  |  |  |  |  |  |
| Antipsychotic free | Reference |  |  |  |  |  |
| Intermittent antipsychotic use | 0.34 | 0.15 | 1.41 | 1.06 | 1.87 | 0.020^*^ |
| Regular antipsychotic use | 0.55 | 0.11 | 1.74 | 1.39 | 2.18 | <0.001^*^ |
| Baseline BMI | 0.10 | 0.01 | 1.10 | 1.09 | 1.12 | <0.001^*^ |
| Baseline FPG | 0.50 | 0.04 | 1.65 | 1.52 | 1.79 | <0.001^*^ |
| Family history of diabetes |  |  |  |  |  |  |
| Without family history of DM | Reference |  |  |  |  |  |
| With family history of DM | 1.55 | 0.16 | 4.72 | 3.42 | 6.51 | <0.001^*^ |
| Marital status |  |  |  |  |  |  |
| Unmarried | Reference |  |  |  |  |  |
| Married | 0.30 | 0.09 | 1.35 | 1.13 | 1.61 | 0.001^*^ |
| Divorced or widowed | 0.21 | 0.12 | 1.23 | 0.97 | 1.57 | 0.090 |
| Educational level |  |  |  |  |  |  |
| Primary schools or below | Reference |  |  |  |  |  |
| Middle school | 0.03 | 0.07 | 1.03 | 0.91 | 1.17 | 0.654 |
| High school or above | -0.06 | 0.10 | 0.94 | 0.78 | 1.14 | 0.545 |
| Occupation |  |  |  |  |  |  |
| Unemployed | Reference |  |  |  |  |  |
| Agricultural workers | -0.43 | 0.23 | 0.65 | 0.42 | 1.02 | 0.060 |
| Other professions | -0.30 | 0.23 | 0.74 | 0.47 | 1.17 | 0.196 |
| Smoking status |  |  |  |  |  |  |
| Nonsmoker | Reference |  |  |  |  |  |
| Former smoker | 0.08 | 0.36 | 1.08 | 0.53 | 2.20 | 0.831 |
| Current smoker | 0.40 | 0.12 | 1.50 | 1.19 | 1.88 | 0.001^*^ |
| Alcohol drinking |  |  |  |  |  |  |
| Never | Reference |  |  |  |  |  |
| Occasionally | -0.16 | 0.17 | 0.85 | 0.60 | 1.19 | 0.348 |
| Frequently | -0.17 | 0.30 | 0.85 | 0.47 | 1.54 | 0.586 |
| Every day | 0.50 | 0.33 | 1.65 | 0.86 | 3.14 | 0.129 |
| Dietary pattern |  |  |  |  |  |  |
| Plant-based diet | Reference |  |  |  |  |  |
| Balanced plant- and animal-based diet | -0.32 | 0.10 | 0.73 | 0.60 | 0.88 | 0.001^*^ |
| Animal-based diet | -0.34 | 0.23 | 0.71 | 0.46 | 1.11 | 0.130 |
| Physical activity |  |  |  |  |  |  |
| Seldom | Reference |  |  |  |  |  |
| Occasionally | -0.12 | 0.20 | 0.89 | 0.60 | 1.32 | 0.555 |
| Once a week | -0.12 | 0.12 | 0.89 | 0.71 | 1.12 | 0.322 |
| Every day | -0.36 | 0.08 | 0.70 | 0.59 | 0.83 | <0.001^*^ |
| Insight rating |  |  |  |  |  |  |
| Complete | Reference |  |  |  |  |  |
| Incomplete | -0.36 | 0.07 | 0.70 | 0.60 | 0.80 | <0.001^*^ |
| Absent | -0.34 | 0.26 | 0.72 | 0.43 | 1.20 | 0.203 |
| Risk rating |  |  |  |  |  |  |
| Level 0 | Reference |  |  |  |  |  |
| Level 1 | 0.04 | 0.18 | 1.04 | 0.74 | 1.47 | 0.826 |
| Level 2 or higher | 0.03 | 0.38 | 1.03 | 0.49 | 2.18 | 0.939 |
| Baseline serum albumin | 0.00 | 0.00 | 1.00 | 1.00 | 1.00 | 0.676 |
| Baseline serum creatinine | 0.00 | 0.00 | 1.00 | 1.00 | 1.00 | 0.455 |
| Baseline serum nitrogen | -0.01 | 0.00 | 0.99 | 0.99 | 1.00 | 0.059 |

Note: ^*^*P*<0.05. DM = diabetes mellitus; HR = hazard ratio; CI = confidence interval; BMI = body mass index; FPG = fasting plasma glucose.
